# NT1014, a novel biguanide, inhibits ovarian cancer growth in vitro and in vivo

**DOI:** 10.1186/s13045-016-0325-7

**Published:** 2016-09-21

**Authors:** Lu Zhang, Jianjun Han, Amanda L. Jackson, Leslie N. Clark, Joshua Kilgore, Hui Guo, Nick Livingston, Kenneth Batchelor, Yajie Yin, Timothy P. Gilliam, Paola A. Gehrig, Xiugui Sheng, Chunxiao Zhou, Victoria L. Bae-Jump

**Affiliations:** 1 Department of Gynecologic Oncology, Shandong Cancer Hospital and Institute, Jinan, People’s Republic of China; 2 Division of Gynecologic Oncology, University of North Carolina, Chapel Hill, NC USA; 3 Department of Surgical Oncology, Shandong Cancer Hospital and Institute, Jinan, China; 4 NovaTarg Therapeutics, Research Triangle Park, Durham, NC 27709 USA; 5 School of Medicine and Life Sciences, University of Jinan, Shandong Academy of Medical Sciences, Jinan, People’s Republic of China; 6 Lineberger Comprehensive Cancer Center, University of North Carolina at Chapel Hill, Chapel Hill, NC 27599 USA

**Keywords:** Biguanide, NT1014, Ovarian cancer, Small compound, AMPK

## Abstract

**Background:**

NT1014 is a novel biguanide and AMPK activator with a high affinity for the organic cation-specific transporters, OCT1 and OCT3. We sought to determine the anti-tumorigenic effects of NT1014 in human ovarian cancer cell lines as well as in a genetically engineered mouse model of high-grade serous ovarian cancer.

**Methods:**

The effects of NT1014 and metformin on cell proliferation were assessed by MTT assay using the human ovarian cancer cell lines, SKOV3 and IGROV1, as well as in primary cultures. In addition, the impact of NT1014 on cell cycle progression, apoptosis, cellular stress, adhesion, invasion, glycolysis, and AMPK activation/mTOR pathway inhibition was also explored. The effects of NT1014 treatment in vivo was evaluated using the K18 − gT121^+/−^; p53^fl/fl^; Brca1^fl/fl^ (KpB) mouse model of high-grade serous ovarian cancer.

**Results:**

NT1014 significantly inhibited cell proliferation in both ovarian cancer cell lines as well as in primary cultures. In addition, NT1014 activated AMPK, inhibited downstream targets of the mTOR pathway, induced G1 cell cycle arrest/apoptosis/cellular stress, altered glycolysis, and reduced invasion/adhesion. Similar to its anti-tumorigenic effects in vitro, NT1014 decreased ovarian cancer growth in the KpB mouse model of ovarian cancer. NT1014 appeared to be more potent than metformin in both our in vitro and in vivo studies.

**Conclusions:**

NT1014 inhibited ovarian cancer cell growth in vitro and in vivo, with greater efficacy than the traditional biguanide, metformin. These results support further development of NT1014 as a useful therapeutic approach for the treatment of ovarian cancer.

## Background

Ovarian cancer is a highly fatal disease that is estimated to cause 14,240 deaths in 2016 in the USA alone [1, 2]. Despite advances in treatment, the 5-year overall survival for ovarian cancer is approximately 40 %. While 80 % of patients will initially respond to cytoreductive surgery and platinum-based combination chemotherapy, the vast majority of women with advanced ovarian cancer will ultimately develop a recurrence and chemo-resistant disease. Thus, there is an urgent need to develop novel therapies for this deadly disease [3, 4].

Obesity is associated with increased risk and worse outcomes for ovarian cancer [5]. The Ovarian Cancer Association Consortium reported that a high BMI, at all stages of life, was associated with an increased risk of developing ovarian cancer [6], while a large prospective cohort study and two systematic reviews reported an increased risk of mortality from ovarian cancer in obese patients [7–9]. In addition to obesity, type II diabetes appears to effect ovarian cancer survival. A recent study following 642 cases of ovarian cancer over a 10-year period found that diabetics with ovarian cancer had significantly worse overall survival as compared to non-diabetics, even after multivariable adjustment [10]. In addition, our laboratory has shown that the metabolic effects of obesity promote ovarian cancer progression and aggressiveness in a genetically engineered mouse model of serous ovarian cancer [11].

The biguanide, metformin, is one of the most widely prescribed treatments for type II diabetes. Epidemiological studies suggest that metformin use for the treatment of type II diabetes may reduce the risk of developing ovarian cancer. This reduction in risk may be due to inhibition of cellular proliferation via AMPK-dependent or AMPK-independent pathways and/or by reducing elevated systemic insulin levels [12–14]. Several recent studies have reported that metformin has an ability to inhibit cell proliferation, adhesion, migration, and angiogenesis in ovarian cancer cell lines and mouse models [15–18]. Our laboratory has found that metformin inhibits cell proliferation in a dose-dependent manner in ovarian cancer cell lines and reduces tumor growth in a genetically engineered mouse model of serous ovarian cancer fed with high-fat and low-fat diets (submitted).

Metformin is transported into cells by organic cation transporters (OCTs) 1, 2, and 3. These transporters are expressed at varying levels in different organs including the liver, muscle, ovary, and kidney [19, 20]. OCT1 and OCT3 are highly expressed in epithelial ovarian cancer and ovarian germ cell tumors, respectively [21, 22]. OCT2 is predominantly expressed in the kidney and is responsible for metformin clearance in the urine. Urinary excretion of metformin results in the short half-life of metformin as well as the wide range of peak to trough drug levels seen, particularly in patients with impaired renal function [19, 23]. Recent studies have shown that inhibition of OCT2 activity by the OCT2 inhibitor cimetidine in patients treated with cisplatin resulted in a decreased cisplatin-induced nephrotoxicity by restricting the accumulation of cisplatin in the kidney [24–26]. Thus, development of novel biguanide agents, designed to increase their affinity for OCT1 and OCT3 while minimizing their affinity for OCT2, may result in more potent drugs with a longer plasma half-life than metformin. Biguanides with this profile may have profound effects on metabolic parameters as OCT1 is highly expressed in the liver whereas OCT3 is expressed in the skeletal muscle [19, 27, 28].

We have recently designed, synthesized, and screened approximately 140 biguanides in an attempt to identify compounds with a high affinity for OCT1 and OCT3 and with a reduced activity at OCT2. The biguanide, NT1014, has activity for OCT1 and 3 and reduced potency for OCT2. Moreover, NT1014 at 20 % the dose of metformin was demonstrated to result in activation of AMPK, inhibition of hepatic glucose output in rat hepatocytes, reduced rate of gastric emptying in mice, increased glucose disposal, and glucose-stimulated glucagon-like peptide-1(GLP1) release (data not shown). In the present study, we investigated the potential of NT1014 as a therapeutic agent for ovarian cancer by evaluating the anti-tumor effects of NT1014 as compared to metformin in human ovarian cancer cell lines and a genetically engineered mouse model of serous ovarian cancer.

## Methods

### Cell culture and reagents

Ovarian cancer cell lines, IGROV-1 and SKOV3, were used in these experiments. IGROV-1 cells were grown in RPMI 1640 medium containing 10 % fetal bovine serum. SKOV3 cells were grown in DMEM/F12 medium with 10 % fetal bovine serum. These two cell lines were cultured with 1 % penicillin-streptomycin at 37 °C under a humidified atmosphere containing 5 % CO_2_. NT1014 was provided by NovaTarg Therapeutics (Cary, NC). Metformin, 3-(4, 5-dimethyl-2-thiazolyl)-2, 5-diphenyl-2*H*-tetrazolium bromide (MTT), and RNase A were purchased from Sigma (St. Louis, MO). All antibodies were obtained from Cell Signaling (Danvers, MA). The L-lactate assay kit was bought from Eton Bioscience (San Diego, CA). 2-[*N*-(7-Nitrobenz-2-oxa-1,3-diazol-4-yl)amino]-2-deoxy-d-glucose (2-NBDG), caspase 3 assay kit, and ATP assay kit were bought from AAT Bioquest (Sunnyvale, CA). Annexin V FITC kit was purchased from BioVision (Mountain View, CA). Enhanced chemiluminescence Western blotting detection reagents were purchased from Amersham (Arlington Heights, IL). All other chemicals were purchased from Sigma (St. Louis, MO).

### Cell proliferation assay

The MTT assay was employed to measure cell proliferation. Briefly, the IGROV-1 and SKOV3 cells were seeded in 96-well plates at a density of 4000 cells/well and allowed to attach overnight. The culture medium was replaced with fresh medium containing NT1014 or metformin (from 0.01 to 3000 μM), and cells were incubated for 72 h. After drug treatment, MTT (5 mg/ml) was added to the 96-well plates at 5 μl/well for an additional incubation time of 1 h. The MTT reaction was terminated through the replacement of the media by 100 μl DMSO. The results were determined by measuring the absorbance at 575 nm with a micro-plate reader (Tecan, Morrisville, NC). The effect of NT1014 and metformin was calculated as a relative percentage of control cell growth obtained from DMSO (0.1 %)-treated cells grown in the same 96-well plates. Each experiment was performed in triplicate and repeated three times to assess for consistency of results.

### Cell cycle analysis

The effect of NT1014 on cell cycle progression was measured using Cellometer (Nexcelom, Lawrence, MA). Briefly, the IGROV1 and SKOV3 cells were plated at 2.5 × 10^5^ cells/well in six-well plates and incubated overnight. Plates were then treated with NT1014 (from 0.1 to 1000 μM) for 24 h. The cells were harvested by trypsin digestion and washed with phosphate-buffered saline (PBS), before being re-suspended and fixed in 90 % pre-chilled methanol and stored at −20 °C overnight. The cells were treated with 50 μl RNase A solution (250 μg/ml, 10 mM EDTA) for 30 min at 37 °C and then stained with 50 μl of staining solution (containing 2 mg/ml propidium iodide (Hayward, MA), 0.1 mg/ml azide, and 0.05 % Triton X-100). The final mixture was incubated for 15 min in the dark before being analyzed by Cellometer. The results were analyzed using FCS4 express software (Molecular Devices, Sunnyvale, CA). The experiments were performed in triplicate and repeated three times for assessment of consistency.

### Annexin V assay

The percentage of cells actively undergoing apoptosis was assessed with the annexin V FITC assay kit. The IGROV-1and SKOV3 cells (2 × 10^5^ cells/well) were treated with NT1014 (from 0.1 to 1000 μM) for 24 h. The cells were then collected, washed with PBS, re-suspended in 100 μl of annexin V and propidium iodide (PI) dual-stain solution (0.1 μg of annexin V FITC and 1 μg of PI), and allowed to incubate for 15 min in the dark. The samples were then analyzed via Cellometer. The results were analyzed by FCS4 express software. All experiments were performed in triplicate and repeated three times to assess for consistency of response.

### Cleaved caspase 3 assay

Cleaved caspase 3 was detected using the cleaved caspase 3 activity assay kit. The IGROV-1 and SKOV3 cells were seeded at 6000 cells/well in a 96-well plate for 24 h and then treated with media containing different concentrations of NT1014 (1–1000 μM) for 4 h. We then added 100 μl of caspase 3 assay loading buffer into each well, mixed gently, and incubated the cells for 60 min at room temperature. The fluorescence intensity was measured at an excitation wavelength of 350 nm and an emission wavelength of 450 nm using a plate reader (Tecan). All experiments were performed at least twice to assess for consistency of response.

### ROS assay

The alteration of total production of reactive oxygen species caused by NT1014 was measured using a DCFH-DA fluorescent dye. The IGROV-1 and SKOV3 cells (1.0 × 10^4^ cells/well) were seeded in black 96-well plates. After 24 h, the cells were treated with NT1014 (0.1 to 1000 μM) for 4 h to induce reactive oxygen species (ROS) generation. After the cells were incubated with DCFH-DA (20 μM) for 30 min, the fluorescence was monitored at an excitation wavelength of 485 nm and an emission wavelength of 530 nm using a plate reader (Tecan). All experiments were performed at least twice to assess for consistency of response.

### Adhesion assay

Each well in a 96-well plate was coated with 100 μl laminin-1 (10 μg/ml) and incubated at 37 °C for 1 h. This fluid was then aspirated, and 200 μl blocking buffer was added to each well for 45–60 min at 37 °C. The wells were then washed with PBS, and each plate was allowed to chill on ice. Next, 2.5 × 10^3^ cells were added with PBS to each well, followed by varying concentrations of NT1014. Each plate was then allowed to incubate at 37 °C for 2 h. After this period, the medium was aspirated, and cells were fixed by adding 100 μl of 5 % glutaraldehyde and incubating for 30 min at room temperature. Adherent cells were then washed with PBS and stained with 100 μl of 0.1 % crystal violet for 30 min. The cells were then washed repeatedly with water, and 100 μl of 10 % acetic acid was added to each well. After 5 min of shaking, the absorbance was measured at 570 nm using a micro-plate reader (Tecan). Each experiment was repeated at least twice for consistency of response.

### Invasion assay

Ninety-six-well HTS transwells (Corning Life Sciences, Durham, NC) coated with 0.5–1X BME (Trevigen, Gaithersburg, MD) were used to examine the effect of NT1014 on the ability of ovarian cancer cells to invade. The IGROV-1 and SKOV3 cells (50,000 cells/well) were starved for 12 h and then added in the upper chambers of the wells in 50 μl FBS-free medium. The lower chambers were filled with 150 μl medium with various concentrations of NT1014. The plate was then incubated for 4 h at 37 °C to allow invasion into the lower chamber. After washing the upper and lower chambers with PBS, 100 μl calcein AM solution was added into the lower chamber and incubated at 37 °C for 30–60 min. The lower chamber plate was measured by the plate reader (Tecan) using an excitation wavelength of 485 nm and an emission wavelength of 520 nm. Each experiment was performed at least twice for consistency of response.

### ATP assay

ATP production was detected by using the luminometric ATP assay kit (AAT bioquest, Sunnyvale, CA), following the manufacturer’s instructions. Each well of a 96-well white plate was seeded with 5 × 10^3^ cells and incubated overnight. Wells were then treated with different doses of NT1014 for 24 h. Next, 100 μl of ATP assay solution was added into each well, gently mixed, and allowed to incubate for 20 min at room temperature. The luminescence intensity was measured using the luminometer mode on a plate reader (Tecan). Finally, the measured ATP levels were normalized based on viable cell counts as measured by MTT assay. The experiments were performed in triplicate and repeated three times for consistency of response.

### Lactate production assay

The L-Lactate Assay Kit was used to measure L-lactate production in the medium. Briefly, after we treated cells with different concentrations of NT1014 for 24 h, 10 μl of the culture medium was transferred into a new 96-well plate, and 40 μl of distilled water was added to each well. Each well was mixed with another 50 μl of lactate assay solution, incubated for 30 min at 37 °C without CO_2_. The lactate level was measured at wavelength of 490 nm using a plate reader (Tecan). The experiments were performed in triplicate and repeated twice to assure consistency.

### Glucose uptake assay

The IGROV-1 and SKOV3 cells were seeded into 96-well black plates at 4000 cells/well overnight and then treated with NT1014 under varying concentrations of glucose for 24 h. After treatment, cells were cultured with 2-NBDG (100 μg/ml) in glucose-free medium for 15 min. The 2-NBDG uptake reaction was stopped by removing the medium and washing the cells twice with 200 μl HBSS (Life Technologies Corporation, Grand Island, NY). Fluorescence intensity was measured at an excitation wavelength of 485 nm and an emission wavelength of 530 nm using a plate reader (Tecan). Relative glucose was assayed compared with untreated control. Data were normalized based on the viable cell counts measured by MTT assay. All the experiments were performed in triplicate and repeated three times.

### Western blot analysis

The IGROV-1 and SKOV3 cells were collected at the end of drug treatment, and total protein was extracted using RIPA buffer (Boston Bioproducts, Ashland, MA) supplemented with protease/phosphatase inhibitor. Equal amounts (30 μg) of total protein were loaded and separated by 10–12 % SDS-PAGE and then transferred to a PVDF membrane. The blot was subsequently blocked in 5 % non-fat milk and incubated with a 1:1000 dilution of primary antibodies at 4 °C overnight. The membranes were then washed and incubated with the appropriate secondary antibodies for 1 h at room temperature before development. The bands were developed and quantified using an Alpha Innotech Imaging System (San Leandro, CA, USA). After developing, the membranes were stripped or washed and re-probed using antibodies against total AMPK or pan-S6 and α-tubulin (for all proteins), respectively. The intensity of bands was measured and normalized to α-tubulin. Each experiment was repeated at least twice for consistency of results.

### KpB mouse model

The K18 − gT121^+/−^; p53^fl/fl^; Brca1^fl/fl^ (KpB) mouse model has been described previously in detail [11, 29]. All mice were handled according to protocols approved by UNC-CH Institutional Animal Care and Use Committee (IACUC). The KpB mice were injected with recombinant adenovirus Ad5-CMV-Cre (AdCre, Transfer Vector Core, University of Iowa) into the left ovarian bursa cavity at 6–8 weeks age. The mice were randomly divided into three groups with one group receiving NT1014 oral garage (dose of 75 mg/kg for 4 weeks) daily, one group receiving metformin in oral gavage (75 mg/kg for 4 weeks), and the other group receiving placebo once the ovarian tumor size had reached 0.1 × 0.1 cm in diameter by palpation. Tumor size was monitored twice-weekly using palpation until tumors had grown to a size amenable to caliper measurement. All mice were euthanized after 4 weeks of NT1014 and placebo treatment. Tumor volume was calculated using the following: (width^2^ × length)/2. Tumor tissues and blood samples were collected for immunohistochemical (IHC) staining and VEGF assay.

### Immunohistochemical analysis

Five micrometer paraffin sections were prepared from the KpB mice tumors and were used for IHC analysis. Staining procedures were performed at the IHC Mice Core Facility at UNC. The following primary antibodies were used: Ki-67, phosphorylated-AKT, phosphorylated-AMPK, phosphorylated-S6, and MMP9. Further processing was carried out using ABC-Staining Kits (Vector Labs, Burlingame, CA) and hematoxylin. IHC slides were scanned by Aperio and scored by ImageScope software (Vista, CA).

### Statistical analysis

Data is expressed as mean ± SEM. Data was compared using two-tailed Student’s *t* test, and *p* < 0.05 was considered significant. Data was analyzed using Prism (GraphPad Software, La Jolla, USA).

## Results

### NT1014 has high affinity for OCT1 and OCT3

**Fig. 1:**
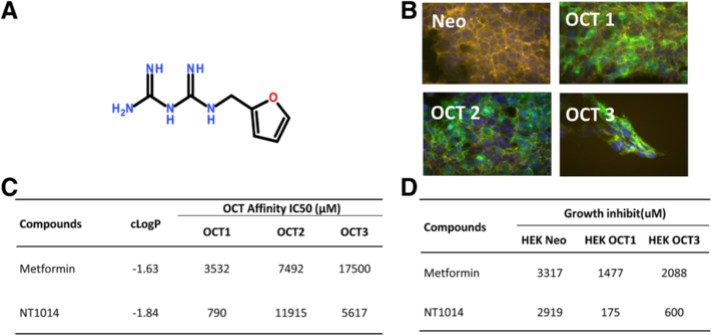
NT1014 has high affinity for OCT1 and OCT3. Molecular structure of NT1014 (**a**). HEK293 cells were stably transfected with hOCT1, 2, and 3 (**b**). *Blue color* represents nuclei. Affinity for OCT1, OCT2, and OCT3 after treatment of NT1014 or metformin (**c**). MTT assay were used to assess the growth inhibition by NT1014 and metformin in HEK293 cells transfected with OCT1 and OCT3 (**d**). *p < 0.05 and **p < 0.01

NT1014 was designed, synthesized, and identified in targeted screening using the ethidium bromide uptake assay in HEK293 cells (Fig. 1a). The cells were stably transfected with hOCT1, 2, or 3, and the uptake of metformin and NT1014 was measured in each cell type and in neo (control) cells at 37 °C for 2.5 min (Fig. 1b). NT1014 had a higher affinity for OCT1 and OCT3 and a reduced activity for OCT2 compared to metformin (Fig. 1c). In addition, MTT assays indicated that NT1014 treatment resulted in a 16-fold increase in growth inhibition (IC50 value) in HEK293 cells stably transfected with OCT1 compared to HEK293 control cells and a 5-fold increase in growth inhibition (IC50) in HEK293 cells stably transfected with OCT3 (Fig. 1d). These results when compared to metformin demonstrate the improved affinity of NT1014 for OCT1 and OCT3 and the reduced affinity for OCT2.

### NT1014 inhibits cell proliferation in ovarian cancer cells

**Fig. 2:**
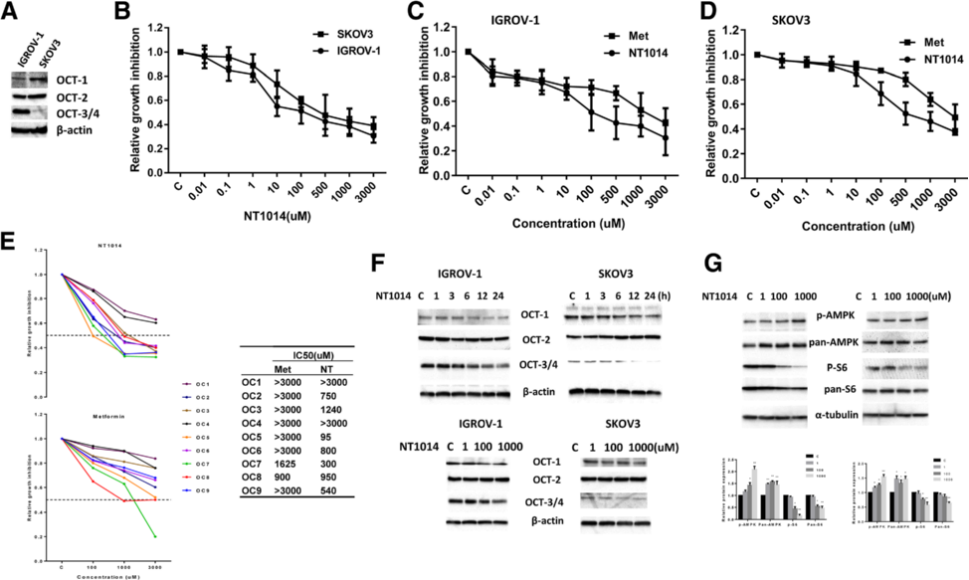
NT1014 inhibited cell proliferation in ovarian cancer cells. The expression of OCT1, OCT2, and OCT3/4 in the IGROV-1 and SKOV3 cell lines was detected by Western blotting (**a**). The IGROV-1 and SKOV3 cells were incubated with NT1014 (from 0.01 to 3000 μM) in 96-well plates for 72 h, and cell proliferation was assessed by MTT assay (**b**). MTT assay also used to compare the growth inhibition of metformin and NT1014 in both cells (**c**, **d**). NT1014 inhibited cell growth in nine primary cultures of ovarian cancer (**e**). IGROV-1 and SKOV3 cells were treated with NT1014 in a time course fashion or treated with different doses for 24 h, and expression of OCT1, OCT2 and OCT3/4 was determined by Western blotting. Equal loading was confirmed with an anti-β-actin antibody (**f**). NT1014 increased the expression of phos-AMPK and decreased phos-S6 expression in both cells after 24 h of treatment (**g**). *p < 0.05 and **p < 0.01

The IGROV-1 and SKOV3 ovarian cancer cell lines were found to express OCT1, OCT2, and OCT3, by Western blotting analysis (Fig. 2a). Using the MTT cytotoxicity assay, the IGROV-1 and SKOV3 ovarian cancer cell lines were found to have a progressive decrease in cell viability with increasing concentrations of NT1014 for 72 h (Fig. 2b). The IC50 values for the IGROV-1 and SKOV3 cells were 200 and 450 μM, respectively, suggesting that IGROV-1 cells are more sensitive to NT1014 than the SKOV3 cells. Subsequently, we compared the effect of NT1014 and metformin on cell proliferation in both cell types. We observed that NT1014 and metformin at low doses (0.01 to 10 μM) produced the same inhibitory effects on cell proliferation. However, NT1014 at high doses was found to increase the growth inhibition in both cells compared to metformin at the same dosages, which the IC50 values were lower for NT1014 than metformin (Fig. 2c, d). To further determine growth inhibitory function of NT1014, we examined the effect of NT1014 and metformin in primary cultures of human ovarian cancers. Cell proliferation in the nine primary cell cultures was assessed by MTT assay after exposure to NT1014 or metformin for 72 h. All nine primary cultures responded to NT1014 or metformin treatment. Lower IC50 values were found for NT1014 as compared to metformin in 6/9 of the primary cultures (Fig. 2e). These results suggest that NT1014 may have improved potency over metformin in inhibition of cell proliferation.

To investigate the effects of NT1014 on expression of OCT1, OCT2, and OCT3/4 in the IGROV-1 and SKOV3 cells, we treated both cell lines with 500 μM NT1014 in a time course fashion. NT1014 decreased OCT1 and OCT3/4 expression in both cell lines, with the greatest effects seen in both cell lines after 24 h of exposure to NT1014. NT1014 did not affect OCT2 expression in the IGROV-1 cells and slightly increased OCT2 expression after 6 h of treatment in the SKOV3 cells. Next, we treated the cells with different doses of NT1014 for 24 h and evaluated the effect of different concentrations of NT1014 on the expression of the OCTs. The level of OCT1 and OCT3/4 protein expression in both cells was decreased in a dose-dependent manner (Fig. 2f). To ascertain whether the effect of NT1014 was mediated by AMPK pathway, we characterized the effect of NT1014 on downstream targets of the AMPK/mTOR/S6 pathway. NT1014 increased phosphorylation of AMPK and decreased phosphorylation of S6 expression in both cell lines after 24 h of treatment (Fig. 2g).

### NT1014 induced cell cycle G1 arrest and cellular apoptosis

**Fig. 3:**
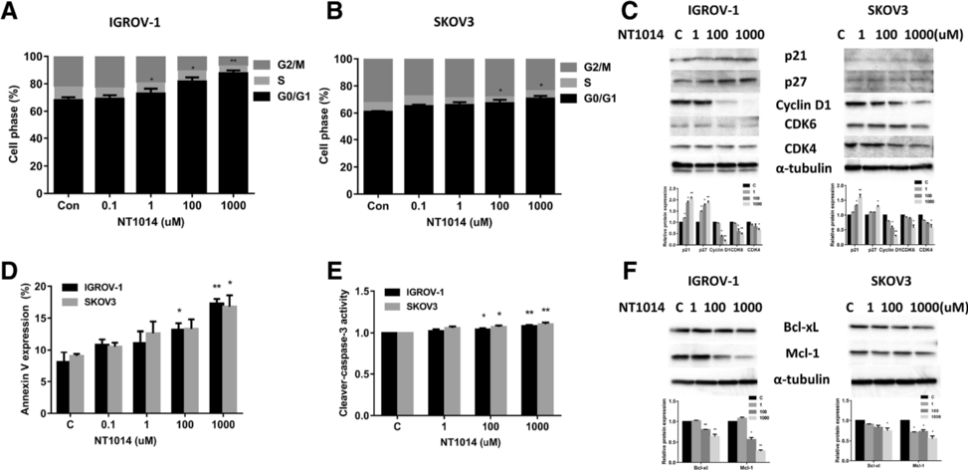
NT1014 induced cell cycle G1 arrest and cellular apoptosis. The IGROV-1 and SKOV3 cell lines were treated with NT1014 for 24 h. Cell cycle progression was analyzed by Cellometer. NT1014 induced G0/G1 cell cycle arrest and reduced S phase in a dose-dependent manner in both cell lines (**a**, **b**). The effect of NT1014 on cell cycle-related proteins (p21, p27, cyclin D1, CDK4, and CDK6) was assessed by Western blotting (**c**). Both cells were treated with varying doses of NT1014 for 24 h, and cell apoptosis was examined by an annexin V FITC assay via Cellometer. NT1014 significantly increased annexin V expression in a dose-dependent manner (**d**). The activity of cleaved caspase 3 was detected by ELISA assay after treatment of NT1014 for 4 h (**e**). Western blotting showed that NT1014 decreased the expression of BCL-xL and Mcl-1 in the IGROV-1 and SKOV3 cell lines after exposure to NT1014 for 24 h (**f**). α-tubulin used as a loading control. *p < 0.05 and **p < 0.01

The effects of NT1014 on cell cycle progression and apoptosis were evaluated in the IGROV-1 and SKOV3 cell lines. The cells were treated with NT1014 at varying concentrations for 24 h, and Cellometer was used to analyze the cell cycle. NT1014 treatment resulted in G0/G1 cell cycle arrest and reduced S phase in a dose-dependent manner in both cell lines (Fig. 3a, b). While the percent of cells in G1 phase increased from 68.2 to 87.7 %, the S phase cell population decreased from 9.6 to 5.5 % with increasing concentrations of NT1014 in the IGROV-1 cells. NT1014 also increased the percent of cells in G1 phase by 9.7 % with concordant reduction of S phase cells by 2.2 % at the dose of 1000 mM in the SKOV3 cell line.

To further characterize NT1014’s effects on cell cycle arrest, cell cycle-related proteins were analyzed in NT1014-treated IGROV-1 and SKOV3 cells. Western blotting results showed that NT1014 down-regulated cyclin D1 and CDK4 and CDK6 protein expression and upregulated cell cycle inhibitor p21 and p27 expression in both cell lines (Fig. 3c). To confirm whether the growth inhibition of ovarian cancer cells was related to apoptosis, the apoptotic effect of NT1014 was evaluated in the IGROV-1 and SKOV3 cells by annexin V FITC stain analysis. Annexin V FITC detects the phospholipid phosphatidylserine (PS) translocation from the inner (cytoplasmic) leaflet of the cell membrane to the external surface in early apoptotic cells. The apoptotic cell population significantly increased in a dose-dependent manner in both cell lines after 24 h of exposure to NT1014 (Fig. 3d). We next determined whether the mitochondrial apoptosis pathway which leads to caspase activation and induces cell death was involved in NT1014-induced apoptosis in the ovarian cancer cell lines. We treated both cell lines with increasing concentrations of NT1014 for 4 h, and the activity of cleaved caspase 3 was detected by ELISA assay. A dose-dependent increase in the activity of cleaved caspase 3 was found in both cell lines in response to NT1014 (Fig. 3e). Furthermore, NT1014 produced a decrease in protein expression of BCL-XL and MCL-1 in a dose-dependent manner after treatment with NT1014 for 24 h in both cell lines (Fig. 3f). These results suggest that NT1014 inhibits cell proliferation through the induction of mitochondrial apoptosis and cell cycle G1 arrest in ovarian cancer cells.

### NT1014 induces cellular stress in ovarian cancer cells

**Fig. 4:**
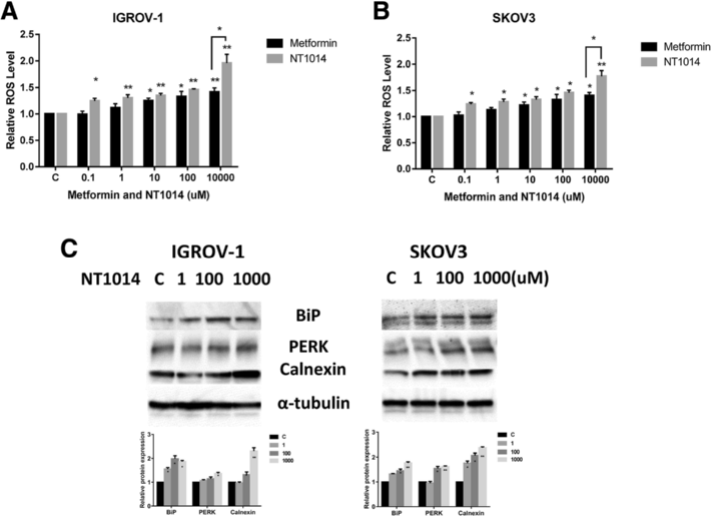
NT1014 induced cellular stress in ovarian cancer cells. The IGROV-1 and SKOV3 cells were treated with NT1014 and metformin at the indicated doses for 4 h, and the ROS production was determined using the DCFH-DA assay. NT1014 increased the ROS level in a dose-dependent manner (**a**, **b**). The expression of cellular stress proteins (PERK, Bip, and calnexin) was detected by Western blotting after treatment of NT1014 for 24 h (**c**). *p < 0.05 and **p < 0.01

ROS have been implicated in the cellular response to stress and are involved in the mediation of apoptosis via mitochondrial DNA damage [20]. Metformin has been shown to induce cell stress in different types of cancer [30]. To investigate the involvement of oxidative stress in the anti-proliferative effect of NT1014, intracellular ROS levels were examined using the ROS fluorescence indicator DCF-DA. NT1014 and metformin (0.1–1000 μM) significantly increased ROS production in a dose-dependent manner in the IGROV-1 and SKOV3 cells after 4 h of treatment (Fig. 4a, b). In addition, NT1014 significantly increased ROS levels in both cells compared to metformin at dose of 1000 μM. We next examined the alternations of endoplasmic reticulum (ER) stress-related markers after 24 h treatment of NT1014 in both cell lines. Our Western blotting results showed that NT1014 significantly induced the protein expression of Bip, PERK, and calnexin in a dose-dependent manner (Fig. 4c). These results indicate that an increase in ROS production and ER stress might also be involved in the anti-tumorigenic effects of NT1014 in ovarian cancer cells.

### NT1014 inhibits cell adhesion and invasion in ovarian cancer cells

**Fig. 5:**
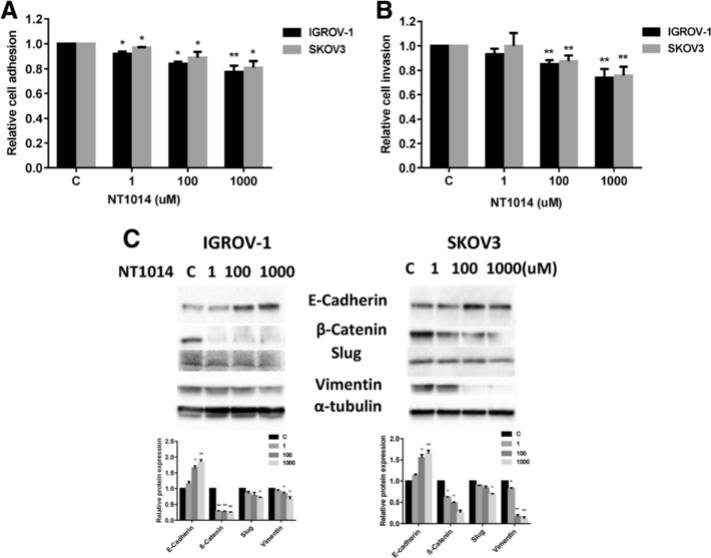
The effect of NT1014 on adhesion and invasion in ovarian cancer cells. The IGROV-1 and SKOV3 cells were cultured for 24 h and then treated with NT1014 (1–1000 μM) in a laminin-coated 96 well plate or BME-coated 96 transwell plate for 2 or 24 h to assess adhesion and invasion in a plate reader. The data represents relative inhibition in each cell line (**a**, **b**). The expression of E-cadherin, β-catenin, Slug, and vimentin were analyzed by Western blotting (**c**). *p < 0.05 and **p < 0.01

In vitro adhesion and invasion assays were performed to evaluate the effect of NT1014 on metastatic activity. Cell adhesion assays were performed using laminin-1 as an adhesion substrate. NT1014 (100 and 1000 μM) treatment of the IGROV-1 and SKOV3 cells for 2 h showed a significant reduction in adhesion to laminin-1 compared with untreated control (17–24 % in IGROV-1 cells and 10–18 % in SKOV3 cells, *p* < 0.05) (Fig. 5a). Both cell lines were again treated with NT1014 at different concentrations for 24 h to determine the effect of NT1014 on cell invasion. NT1014 (100 and 1000 μM) significantly decreased cell invasion activity after 24 h of treatment (15–28 % in IGROV-1 cells and 11–23 % in SKOV3 cells, *p* < 0.05), as determined by the transwell invasion assay (Fig. 5b).

Cell adhesion and invasion are mediated by a variety of membrane proteins as well as modulation of cytoskeletal assembly. To further analyze the effect of NT1014 on cell motility and migration of ovarian cancer cells, the levels of expression of E-cadherin, β-catenin, Slug, and vimentin were analyzed by Western blot. After 24 h of treatment, NT1014 increased expression of E-cadherin and decreased expression of β-catenin, Slug, and vimentin (Fig. 5c). Collectively, these results demonstrate that NT1014 inhibits the adhesion and invasion of ovarian cancer cells.

### The effect of NT1014 on glycolytic metabolism

**Fig. 6:**
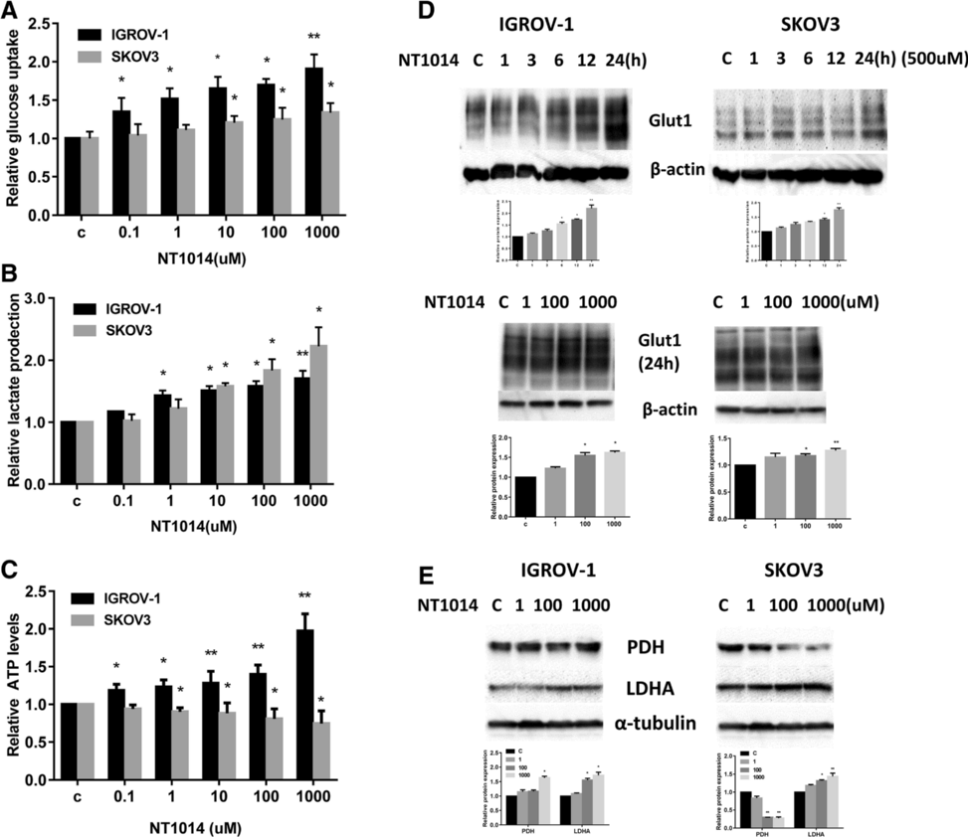
The effect of NT1014 on glycolytic metabolism in ovarian cancer cells. The IGROV-1 and SKOV3 cells were incubated with NT1014 in concentrations of up to 1000 μM for 24 h. The glucose uptake (**a**), lactate level (**b**), and cellular ATP level (**c**) were assayed. NT1014 increased glucose uptake and lactate production. NT1014 treatment resulted in a decrease in ATP production in the SKOV3 cells and an increase in ATP in the IGROV-1 cells (**c**). The expression of Glut1, LDHA, and PDH was measured by Western blotting after treatment with NT1014 for 24 h (**d**, **e**). *p < 0.05 and **p < 0.01

It is well documented that cancer cells undergo a metabolic shift to adapt and survive under harsh environments by enhancing aerobic glycolysis (i.e., the Warburg effect). Cancer cells exhibit increased expression of glucose transporters as a means to enhance glucose uptake, which in turn increases the rate of glycolytic ATP production and ultimately leads to enhanced tumor growth [31]. In order to investigate whether NT1014 affects glycolysis in ovarian cancer cells, the IGROV-1 and SKOV3 cells were incubated with NT1014 in concentrations up to 1000 μM for 24 h. The cellular ATP level, as well as glucose uptake and lactate level, was assayed. NT1014 increased glucose uptake and lactate production in both ovarian cancer cell lines (Fig. 6a, b). Compared to control cells, treatment with NT1014 caused a time-dependent increase in Glut1 expression in both cell lines, as well as a concentration-dependent increase in IGROV-1 cells, suggesting that NT1014 stimulates glycolytic activity (Fig. 6d). Interestingly, NT1014 treatment resulted in a decrease in ATP production in the SKOV3 cells and an increase in ATP in the IGROV-1 cells (Fig. 6c).

To validate the causal relationship between ATP levels and glycolytic activity, we next examined the effect of NT1014 on glycolytic pathway. The expression of pyruvate dehydrogenase (PDH), a critical regulator of transforming pyruvate into acetyl-CoA, and lactate dehydrogenase (LDHA), a key enzyme of converting pyruvate into lactate, were analyzed after incubation with NT1014 for 24 h. We observed increased LDHA protein expression in both cell lines after 24 h of treatment (Fig. 6e), suggesting a direct effect of NT1014 on glucose metabolism and enhanced activity of the glycolytic pathway in the ovarian cancer cells. In addition, we also found that PDH expression was elevated in the IGROV-1 cells and was decreased in the SKOV3 cells after 24 h of treatment, suggesting that the function of complex I in SKOV3 cells compared to IGROV-1 cells was more profoundly influenced by NT1014. Given that biguanides target complex I and subsequently increase glycolytic activity in cancer cells, the differential metabolic reactions of NT1014 on the glycolytic pathway and complex I suggest that ovarian cancer cells have a different metabolic state in response to NT1014 compared to other biguanides such as metformin.

### NT1014 decreased tumor growth in the KpB serous ovarian cancer mouse model

**Fig. 7:**
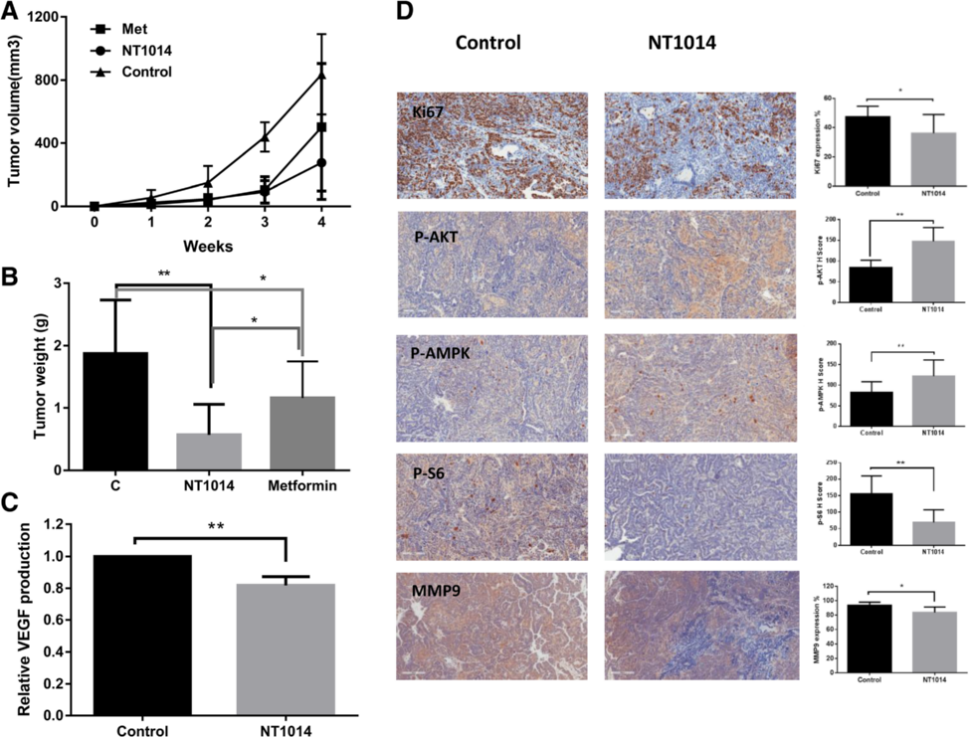
The effect of NT1014 on ovarian tumor growth in the KpB serous ovarian cancer mouse model. Thirty KpB mice were divided into three groups and treated with NT1014 and metformin (oral gavage, 75 mg/kg/day, 6 times/ week) or placebo for 4 weeks. The graph shows weekly tumor volumes for each group (**a**) and tumor weight after treatment (**b**). The level of VEGF in NT1014 group was significantly lower than that in control group (**c**). The changes of Ki67, phos-AKT, phos-AMPK, phos-S6, and MMP9 were assessed by immunohistochemistry in the ovarian cancer tissues. The expression of Ki-67, phos-S6 and MMP9 was significantly reduced and phos-AKT and phos-AMPK was increased in the NT1014 treatment group compared with the control group (**d**). *p < 0.05 and **p < 0.01

The in vivo anti-tumor efficacy of NT1014 was evaluated in the KpB serous ovarian cancer mouse models. The KbB mice were divided into three groups (*n* = 15/group) and were treated with NT1014 and metformin (75 mg/kg/day, 6 times/week) or placebo for 4 weeks. Tumor growth during the treatment period was monitored using twice weekly palpation. NT1014 or metformin was well tolerated. The mice showed no overt signs of toxicity and maintained normal activities throughout treatment. Twice-weekly measurements yielded no changes in blood glucose or mouse weight during NT1014 and metformin treatment (data not shown). After 4 weeks of treatment, the mice were euthanized, and the ovarian tumors were removed, photographed, and weighed. Both NT1014 and metformin resulted in significant suppression of tumor growth relative to the control. NT1014 showed more significant inhibition in tumor growth compared to metformin at the same dose, as evidenced by a decrease in tumor weight of approximately 70 % in NT1014 group and 46 % in metformin (*p* < 0.05) (Fig. 7a, b).

To further investigate the anti-tumor activity and mechanism of NT1014 in vivo, the expression of Ki-67, phosphorylated (phos)-AKT, phos-AMPK, phos-S6, and MMP9 in the ovarian tumor tissues was evaluated by IHC. Consistent with our results in vitro, the expression of phos-AMPK and phos-AKT was induced in the mice treated with NT1014, whereas NT1014 reduced the levels of phos-S6 in the treated mice but not in the untreated mice (Fig. 7d). These findings suggest that NT1014, like other biguanides, inhibits tumor growth of ovarian cancer in vivo via AMPK activation and inhibition of the mTOR pathway. Additionally, Ki-67 and MMP9 expression were significantly reduced following NT1014 treatment compared to the untreated controls (Fig. 7d). Serum VEGF levels were measured by ELISA assay at the end of the treatment. Mean VEGF level in treated group was significantly lower than that in control group (Fig. 7c). These results further support the role of NT1014 in the inhibition of tumor adhesion and invasion in ovarian cancer in vivo.

## Discussion

NT1014 was designed as a novel AMPK activator with high affinity for OCT1 and OCT3. We find that NT1014 is a potent anti-tumorigenic agent that suppresses ovarian cancer cell proliferation and in vivo ovarian tumor growth through activation of AMPK. In addition, our results demonstrate that NT1014 inhibits cell proliferation and tumor growth with higher potency than metformin at similar doses in both ovarian cancer cell lines and in the KpB mouse model. Along with AMPK-induced inhibition of mTOR signaling which has been shown to trigger the anti-tumorigenic activities of metformin, NT1014 interferes with multiple AMPK-dependent downstream signaling pathways that regulate survival, energy metabolism, oxidative stress, and cell migration in ovarian cancer cells.

Cell proliferation assays revealed a dose-dependent inhibition of cell growth by NT1014 in both human ovarian cancer cell lines tested. The drug concentrations required to induce growth inhibition are much lower compared to metformin in the treatment of ovarian cancer cells in vitro. In addition, NT1014 treatment (75 mg/kg) led to profound inhibition of ovarian tumor growth in vivo and had increased efficacy compared to the same dose of metformin (58 versus 33 %). Moreover, the data support that NT1014 binds to OCT1 with higher affinity than metformin, giving it the potential to more effectively enter OCT1-expressing ovarian cancer cells. Recent studies using metformin and AICAR (pharmacological AMPK activators) have confirmed their ability to induce apoptosis and cell cycle arrest in a variety of cancer cell types [31, 32]. Our data showed that induction of apoptosis and cell cycle G1 arrest are key components of the anti-tumorigenic effects of NT1014 in ovarian cancer cells, as evidenced by induced expression of annexin V, p27, and p21 as well as reduction of BCL, Mcl-1, cyclin D1, and CDK expression. These effects may be the result of activation of AMPK and direct inhibition of the mTOR signaling pathway, given that NT1014 treatment increased phosphorylation of AKT in the KpB mouse model.

The underlying mechanism responsible for NT1014 as well as metformin-induced growth inhibition has not been entirely defined. Numerous studies have demonstrated that metformin significantly inhibits cell proliferation through activation of the AMPK pathway in ovarian cancer cells; furthermore, long-term use of metformin has been associated with decreased risk of ovarian cancer and improved outcomes in patients with or without diabetes [12, 14, 33, 34]. Together, these findings suggest that AMPK is an ideal target for the prevention and treatment of ovarian cancer. Recent reports have shown that AMPK activation by metformin is associated with increased oxidative stress leading to upregulated cell cycle arrest and induction of apoptosis in breast cancer and leukemia [35, 36]. The level of oxidative stress correlated to metformin-dependent apoptosis induction in breast cancer [37]. A dose-dependent increase in reactive oxygen species formation with NT1014 treatment was found in this study. Similar NT1014 treatment at different concentrations resulted in an increase in expression of PERK, Bip, and calnexin, which are markers of oxidative stress associated with apoptosis [36]. Our data not only confirms that NT1014 induces cell cycle arrest and apoptosis but also demonstrates that NT1014 induces oxidative stress in the endoplasmic reticulum of treated ovarian cancer cells. The mechanism of apoptotic death may be triggered by the inability of ovarian cancer cells to adequately respond to oxidative stress in the endoplasmic reticulum.

Cell migration is a highly complex process, which involves orchestrated dynamic remodeling of the actin cytoskeleton and microtubule network [38]. AMPK has been recently documented to be involved in this process in cancer cells [38]. Pharmacological activation of AMPK by metformin and other biguanides disturbs cancer cell migration and invasion. Several studies have shown that AMPK inhibits cell migration, which could occur through different mechanisms including disruption of the mTOR, TGF-b, Pdlim5, CXCL12, NF-kB, and Akt-MDM2-Foxo3a pathways [38–43]. Thus, it is reasonable to believe that AMPK activity affects directional cell migration by regulating cell epithelial-mesenchymal migration. In this study, our results demonstrated that treatment with NT1014 modifies the phenotype of ovarian cancer cells from a mesenchymal to an epithelial phenotype as evidenced by increased expression of the epithelial marker E-cadherin and decreased expression of the mesenchymal marker Slug. Thus, NT1014 may have an improved ability to inhibit ovarian cancer metastasis and progression through regulation of the epithelial-mesenchymal transition.

One of the principal metabolic alternations related to cell proliferation in tumors is the upregulation of aerobic glycolytic metabolism [44]. Activating AMPK by different agonists results in differential effects on glycolytic metabolism in cancer cells [45]. Metformin is believed to have an inhibitory effect on mitochondrial oxidative phosphorylation via inhibiting respiratory complex I, thus boosting glycolysis as a compensation mechanism. The effects of NT1014 on glycolysis in ovarian cancer cells parallel those reported for metformin in other types of cancer [45–49]. NT1014 exposure led to an increase in phosphorylation of AMPK and glucose uptake consistent with an increase in glycolysis, characterized by increased lactate production and increased levels of LDHA. Emerging evidence has confirmed that metformin effectively reduces mitochondrial ATP production [49, 50]. In contrast, we found an increase in ATP levels with increased PDH expression in IGROV-1 cells, but a decrease in ATP levels and PDH expression in SKOV3 cells, after treatment of NT1014 for 24 h, despite phosphorylation of AMPK in both cell lines after 12 h of treatment. These results suggest that increased ATP production in IGROV-1 cells via increased PDH expression and acetyl-CoA represents a mechanism that partially compensates for the NT1014-associated decrease in ATP production by oxidative phosphorylation. Therefore, NT1014 may possess an alternative mechanism to regulate glucose metabolism and inhibit cell proliferation compared to metformin in ovarian cancer cells.

## Conclusions

In conclusion, the results from this study show that NT1014 is a novel, orally bioavailable, and well-tolerated AMPK activator with a high affinity for OCT1 and OCT3. NT1014 causes significant inhibition of ovarian cancer cell proliferation in vitro and has anti-tumorigenic activity in vivo against ovarian cancer through modulation of multiple signaling pathways associated with cancer cell survival, metabolism, and progression. These results show promise for the use of NT1014 as a potential anti-cancer agent for the treatment of ovarian cancer. The efficacy of NT1014 in other ovarian cancer cell lines and mouse models, given alone or in combined therapy, will be explored in future studies.

## Abbreviations

2-NBDG: 2-[*N*-(7-Nitrobenz-2-oxa-1,3-diazol-4-yl)amino]-2-deoxy-d-glucose
AdCre: Ad5-CMV-Cre
GLP1: Glucose-stimulated glucagon-like peptide-1
LDHA: Lactate dehydrogenase
MTT: 3-(4, 5-Dimethyl-2-thiazolyl)-2, 5-diphenyl-2*H*-tetrazolium bromide
OCTs: Organic cation transporters
PDH: Pyruvate dehydrogenase
PI: Propidium iodide
